# Usability evaluation of augmented reality glasses for remote support during extracorporeal membrane oxygenation

**DOI:** 10.1016/j.csbj.2025.09.025

**Published:** 2025-09-18

**Authors:** Simon König, Niels Hinricher, Claus Backhaus

**Affiliations:** a FH Münster University of Applied Sciences, Center for Ergonomics and Medical Engineering, Bürgerkamp 3, Steinfurt 48565, Germany; b Technical University Berlin, Institute of Psychology and Ergonomics, Fasanenstraße 1, Berlin 10623, Germany

**Keywords:** Augmented reality (AR), Intensive care medicine, Extracorporeal membrane oxygenation (ECMO), User test, Usability, User acceptance

## Abstract

Extracorporeal membrane oxygenation (ECMO) procedures may result in emergencies requiring the intervention of a perfusionist. However, their arrival results in waiting times that put patients at risk. Hence, nursing staff should be remotely supported using augmented reality (AR) in troubleshooting to improve patient safety. In this study, we aimed to investigate the usability of AR glasses *HMT-1* and *HoloLens 2* for remote support during ECMO and to determine the user acceptance of AR in intensive care. Ten nurses tested both glasses in a user test in randomized order under perfusionist guidance to rectify the three ECMO emergencies: a signal alert, a pump failure and reduced blood flow. An investigator rated their skills using a 3-step scheme, and success rates were calculated from the ratings and compared using the Wilcoxon test (α =.05). AR acceptance was assessed using the System Usability Scale (SUS) and User Experience Questionnaire (UEQ). We recorded 12 and 21 use errors with *HMT-1* and *HoloLens 2,* respectively. However, significantly fewer use errors occurred with the *HMT-1* than with *HoloLens 2* (Z = -1786, p = .046, n = 80) when troubleshooting the pump failure. Most participants rated *HoloLens 2* as more suitable. AR acceptance was rated as good with a SUS score of 72 (± 11). UEQ ratings for *attractiveness*, *dependability*, *stimulation*, *novelty*, and *efficiency* were excellent or good, while *perspicuity* was rated below average. The results indicate that both AR glasses are suitable for remote support; however, improvements to increase efficiency and patient safety in intensive care are necessary.

## Graphical Abstract

**Figure:**
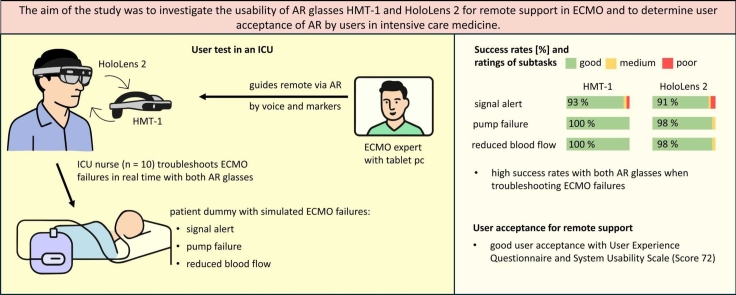

## 1 Introduction

Patients with life-threatening conditions are treated with intensive care medicine. They often present with pulmonary diseases or cardiac dysfunction. In rare, severe cases, extracorporeal membrane oxygenation (ECMO) is required. This high-risk, technically complex therapy supports heart and lung function, and emergencies can be life-threatening within minutes. ECMO is defined as the process of diverting venous blood from a patient to a gas exchange system for the addition of oxygen, removal of carbon dioxide, and subsequent re-infusion to the patient's arterial or venous system. During ECMO, blood is continuously drained from the patient, pumped with either a centrifugal pump or a roller pump, enriched with oxygen and cleansed of carbon dioxide with an artificial membrane, warmed to the body temperature, and returned to the patient [37]. The duration of ECMO therapy varies considerably. While most patients require support for days to weeks, in some cases support has been maintained for several months. Rare reports describe ECMO support lasting over one year as a bridge to transplantation [62]. ECMO is controlled and monitored using a dedicated console.

ECMO is important for intensive care medicine. It has been increasingly used since the outbreak of COVID-19. However, only 12 % of German hospitals can provide this therapy [16], [20], [22], [6] because of the significant effort required to maintain a perfusion department. Furthermore, treatment success is influenced by hospital experience. Friedrichson et al. [22] showed that hospitals that perform more than 30 ECMO therapies per year achieve lower mortality rates. However, this number was reached in only 23 hospitals in Germany, whereas the median was four ECMO therapies per year [22].

Notably, collaboration among physicians, nurses, and perfusionists is required for ECMO therapy [9]. In some ECMO programs, the perfusionist together with a surgical cannulation team is responsible for establishing and maintaining ECMO therapy [7] In the majority of ECMO programs in North America, an ECMO specialist, who may be either a highly trained nurse or respiratory therapist, in collaboration with a surgical cannulation team is responsible for establishing and maintaining the ECMO during therapy. In either scenario, if an ECMO emergency occurs during therapy, the medical staff calls the surgical team and the perfusionist for assistance. However, since the perfusionist's primary responsibility is to operate the heart-lung machine during elective procedures, they are involved during operating hours. Outside of these times, the perfusion department is usually unstaffed, so an on-call service is available for emergencies. Perfusionists typically provide this service from home.

When a nurse reports an emergency, the perfusionist on call or in the operating room first tries to resolve the problem by phone. However, the quality of support depends on the nurse's description of the situation. If telephone assistance is unavailable, the perfusionist must correct the problem on site. Waiting times for the on-call perfusionist to arrive can put the patient at risk. Wait times also occur when the perfusionist cannot leave the operating room immediately or has to travel long distances to the intensive care unit.

To date, these telephone consultations are the most commonly used form of remote support in medicine [39]. Augmented reality (AR) is a technology that can improve remote support [54], as it provides a connection for nurses and perfusionists using special AR glasses and mobile devices (e.g., laptops, tablet PC, or smartphones). The AR glasses have a screen, a camera, and options for audio recording and output. The camera and audio features allow the perfusionist to see and hear conditions at the nurse's location on the mobile device. They can also communicate with the nurse and provide information on the AR glasses display.

The design of these features varies from one model to another. However, the two AR glasses that have been commercially available are *HMT-1* (RealWear Inc., Vancouver, WA, USA) and *HoloLens 2* (Microsoft Corp., Redmond, WA, USA) (Table 1).

**Table 1:** Comparison of the augmented reality glasses HMT-1 and HoloLens 2.

**Feature**		
	***HMT-1***	***HoloLens 2***
Audio	Headphones (In Ear)	Speaker
Screen	LCD (monocular)	See-through (binocular)
Field of view	20°	52°
Control mode	Voice control	Gesture control

The *HMT-1* is designed for industrial use. It has a monocular LCD display in front of the left eye. Visual markers, such as laser pointer dots, can be displayed on the display. However, the display partially covers the real environment. The *HMT-1* is operated hands-free using voice commands. The *HMT-1* uses in-ear headphones for audio playback, which are connected to the glasses via an audio port. At 20°, the field of view (FOV) of the *HMT-1* is relatively narrow compared with that of other AR glasses.

The *HoloLens 2*, for example, has a FOV of 52°. Combined with the binocular see-through display, these glasses make it possible to display holographic content without obscuring the real environment. The *HoloLens 2* is controlled by hand gestures. Instead of selecting menu items by voice, the holographic menu is selected with a finger, just like on a touchscreen. Audio is played through a speaker located on the back of the wearer's head.

AR is already well established in industry for remote support. Common use cases include support during repair or maintenance work or instructing inexperienced employees during assembly processes [21], [29], [44], [55], [67]. The latter is often supplemented with visual instructions [1], [10], [11], [31], [46]. Additionally, isolated studies have investigated the use of this technique for treating soldiers in the field [41], [69]. Miller et al. [48] developed an AR system to improve soldiers’ medical care.

Moreover, the potential of AR in medicine has been discussed previously [15], [33], [66]. However, previous studies have often focused on preoperative planning or consultations during surgical procedures [14], [23], [24], [25], [27], [43], [45], [68]. For example, Sánchez-Margallo et al. [60] used AR before surgery to plan kidney tumor removal. The surgical team rated the technique as helpful. However, the surgeons criticized the *HoloLens 2* for being too heavy to wear for long periods. Furthermore, Andersen et al. [2] developed a system to support inexperienced surgeons, enabling communication with an experienced colleague to create incision paths during surgery [58]. The authors described that the technical requirements resulted from clinical use, such as the precision of the displayed incisions [3]. In addition, Rojas-Muñoz et al. [59] demonstrated the effectiveness of simulated leg surgery.

Despite the positive research results, AR is less established for medical applications. One possible reason for this is the insufficient usability for the often time-critical use cases. Usability describes how effectively, efficiently and satisfactorily a product can be used [35]. It depends on the context of use and the user. Each product must be adapted to the environment, the user group and the work task [4]. To date, only a few studies have investigated the usability of AR glasses [51], [65]. Therefore, knowledge regarding intensive care in this area is lacking.

Hence, this study aimed to investigate the usability of AR glasses *HMT-1* and *HoloLens 2* for remote support in ECMO and determine the acceptance of AR by users in intensive care medicine.

## 2 Methods

### 2.1 Test setup and procedure

**Fig. 1:**
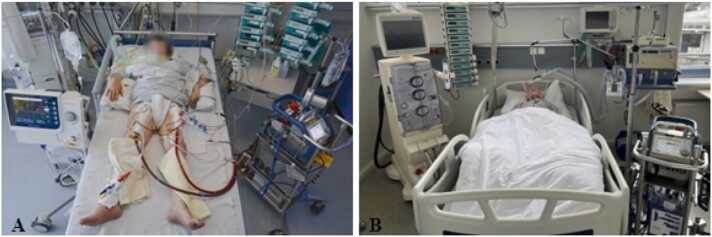
Intensive care patient with extracorporeal membrane oxygenation treatment (A) [56] and test setup (B).

To investigate the usability of the two AR glasses, we performed a user test with nurses. The user test was performed in the patient room in the internal medicine intensive care unit, where the treatment of a patient with ECMO was simulated. An ECMO console (*Rotaflow*, Getinge AB, Sweden) was connected to the patient’s manikin (Fig. 1).

At the beginning of the test, the participants' age, sex, education, work experience, and previous experience with ECMO and AR were recorded. The participants then received standardized training on the two AR glasses. The different concepts of operation were explained. The participants also put on the glasses to adjust them on their own, and they were shown how the two AR glasses display information.

In the test, the participants were required to rectify three ECMO emergencies: a signal alarm, a pump failure, and reduced blood flow. They wore one of the two AR glasses and called a perfusionist. The technician remotely guided the participants in a standardized manner using a tablet PC.

To measure blood flow during ECMO, an ultrasound sensor is placed behind the centrifugal pump, to which contact cream is applied. If the cream dries out, the measurement is no longer possible and an alarm sounds. To clear the alarm, participants had to turn off the ECMO and remove the dried contact cream from the ultrasound sensor. They then applied new contact cream and restarted ECMO.

If the pump failed, participants had to continue the operation manually. To do this, they removed the pump head from the electrical drive and inserted it into a drive with a hand crank. Blood flow was then restored using the hand crank.

If the cause of the reduced blood flow was a kinked tube under the patient's thigh, the participants had to examine the tube, find the kink, and remove it.

**Fig. 2:**
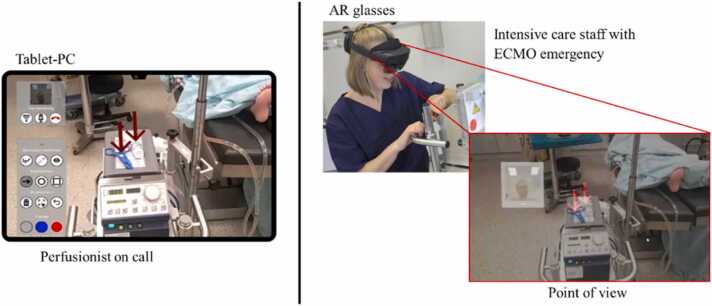
Remote support for troubleshooting on extracorporeal membrane oxygenation.

The perfusionist provided both verbal and visual instructions via markers displayed on the AR glasses. The *HMT-1* used laser pointer dots, whereas the *HoloLens 2* holographically displayed arrows in the FOV. The perfusionist used these markers to highlight relevant components such as the ECMO pump head or tube clamps (Fig. 2).

After solving the three emergencies, the participants changed the AR glasses and solved the emergencies again, also under the guidance of the perfusionist. Five participants started with the *HMT-1* and five with the *HoloLens 2*. For both glasses, the signal alarm was addressed first, then the pump failure, and finally the reduced blood flow.

A test investigator observed the user tests in a separate room. The camera images from the AR glasses were transmitted not only to the perfusionist's tablet PC, but also to the investigator's laptop. In addition, a camera (GoPro Hero 5, GoPro Inc., San Mateo, CA, USA) recorded the test environment. These recordings were also transferred to the laptop.

### 2.2 Evaluation of the usability

**Fig. 3:**
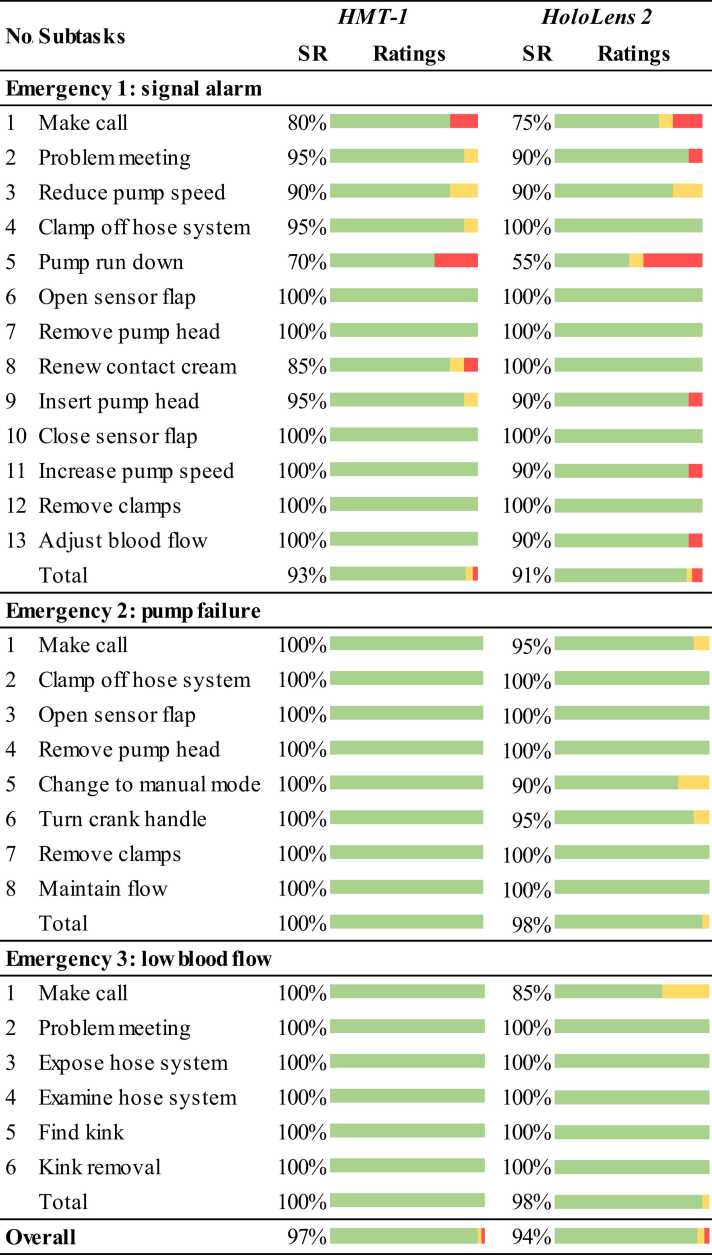
Evaluation of the skills and success rates of the augmented reality glasses.

We divided the emergency rectification into subtasks [17]. Fig. 3 shows the subtasks. The investigator evaluated the participants' abilities to perform each subtask, using a 3-stage scheme for this purpose (Table 2) [26].

**Table 2:** Criteria for evaluating the subtasks.

The relative frequencies of the evaluation levels are visualized in bar charts for each subtask, emergency, and overall. Success rates were calculated following the Nielsen [53] (Eq. 1).

(1) $$\mathrm{SR}[\%]=\;\frac{\sum \mathrm{good}+\;\sum \mathrm{medium}*0,5}{n_{\mathrm{subjects}}*\;n_{\mathrm{subtasks}}}*100$$

A Wilcoxon test (α =.05) was performed to compare the two AR glasses, which was used to test for differences in success rates. Finally, participants completed a survey to rate which of the two AR glasses they preferred.

### 2.3 Survey of user acceptance

**Fig. 4:**
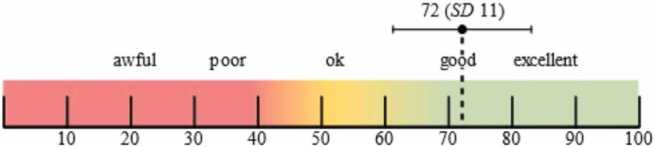
Achieved System Usability Scale Score and interpretation according to Bangor et al. [5].

The participants answered two standardized questionnaires after the test to measure user acceptance: the System Usability Scale (SUS; [12]) and the User Experience Questionnaire (UEQ; [40]). In the SUS, participants’ agreement was rated using 10 alternately formulated items on a 5-point Likert scale (0 = strongly disagree, 4 = strongly agree). Furthermore, each item was scored from 0 to 4 points depending on the level of agreement. A SUS score between 0 (negative) and 100 (positive) was obtained by adding all the points and multiplying by a factor of 2.5. The SUS scores were interpreted using an established benchmark [5] (Fig. 4).

**Fig. 5:**
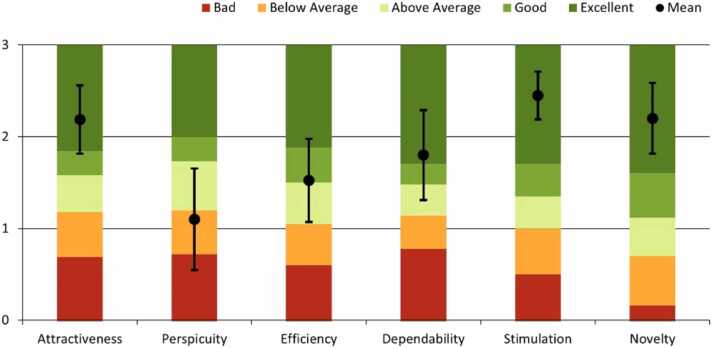
Mean dimension values with 95 % confidence intervals and interpretation according to Schrepp (2017).

We used the UEQ to measure user acceptance based on 26 pairs of contrasting adjectives (good/bad). The participant’s agreement was rated using one of the adjectives on a 7-point Likert scale. Points were awarded for each item between -3 (negative adjective) and +3 (positive adjective) depending on the level of agreement. Each pair of adjectives was assigned one of the following dimensions: *attractiveness*, *perspicuity*, *efficiency*, *dependability*, *stimulation,* or *novelty* [57]. The points were arithmetically averaged for each dimension, and the means were interpreted using an established benchmark [61] (Fig. 5).

## 3 Results

### 3.1 Description of the sample

Ten nurses who had specialized education in intensive care medicine participated in the user test. Eight of the participants were male, and two were female. The mean age and experience of our participants were 39 (± 9) years and 10 (± 9) years, respectively. All participants were familiar with ECMO, but only eight had experience with using the ECMO machine. None of them had any experience with using AR glasses.

### 3.2 Evaluation of the usability

We encountered 12 use errors using *HMT-1* and 21 use errors with *HoloLens 2*. Fig. 3 shows the subtasks, investigator ratings, and success rates (SR) for the two AR glasses.

When the pump failure was rectified, significantly fewer use errors occurred with *HMT-1* than with *HoloLens 2* (Z = -1.786, p = .046, n = 80). Moreover, there were no significant differences in single subtasks, other emergencies, or overall.

Overall, the only use errors that led to a poor rating occurred during the removal of the signal alarm. During *HMT-1* use, two participants needed help from the experimenter to call the perfusionist. In the subsequent emergencies, the calls were made without use errors. Even with *HoloLens 2*, two participants needed help to make the call. In the subsequent emergencies, the *HoloLens 2* caused five more use errors with gesture control.

Three participants did not fully reduce the pump speed when using the *HMT-1* (action step 5). To do this, they had to press down and rotate the rotary push button on the ECMO. Participants released the button before the speed was reduced to zero. This use error also occurred several times with the *HoloLens 2*. The use errors in steps 3, 10, and 11 of the signal alarm are also related to the rotary push button.

Another use error was only observed with the *HMT-1*: two participants applied the contact cream in the wrong place.

In the survey, five participants rated *HoloLens 2,* and one participant rated *HMT-1* as more suitable. However, four of the participants considered both AR glasses equally functional.

### 3.3 Survey of user acceptance

The user survey showed that the acceptance of AR in intensive care medicine was high. A SUS score of 72 (± 11) was achieved, which corresponds to good user acceptance (Fig. 4).

Results from the UEQ showed that the dimensions, such as *attractiveness*, *dependability*, *stimulation* and *novelty,* were rated as excellent and *efficiency* rated as good by the participants. However, the results for the AR were below average in the *perspicuity* dimension (Fig. 5).

## 4 Discussion

### 4.1 Evaluation of the usability

This was the first time the usability of AR glasses was assessed for remote support during ECMO in a semi-controlled study. The high SR showed that both AR glasses are suitable for troubleshooting ECMO emergencies. However, the participants achieved a significantly high success rate with *HMT-1* in rectifying pump failure (Emergency 2). Nevertheless, the majority preferred *HoloLens 2*, probably because it has a larger FOV. This has been identified as an important acceptance criterion in previous studies [19], [28], [49].

**Fig. 6:**
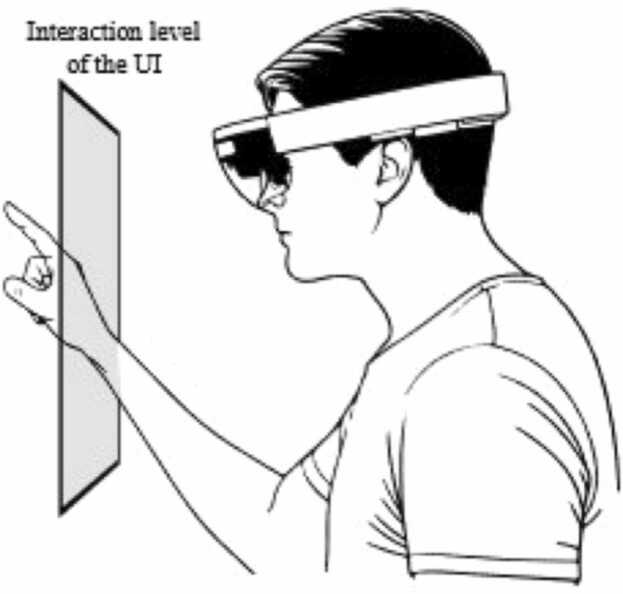
Incorrect assessment of the distance to the virtual user interface.

Furthermore, gesture control increasingly led to errors when *HoloLens 2* was tested. The participants used a virtual user interface (UI) as control. To achieve this, the AR glasses superimposed the UI on the participants’ FOV. However, the participants often estimated the distance between themselves and the UI to be too large. In addition, they often reached too far away when trying to interact with the UI (Fig. 6).

However, Benedict et al. [8] reported that gesture control can be achieved easily. In our study, all participants were able to start the call independently during the second and third emergency. Nevertheless, fewer errors occurred when using voice control with *HMT-1*. As the *HoloLens 2* can also be operated using voice control, this should be implemented in addition to gesture control to reduce use errors.

The participants often positioned the display above or below the eye when testing the *HMT-1* to avoid restricting their FOV. This makes it difficult to see the UI and the visual markers (laser pointer dots) placed on the ECMO components by the perfusionist. Therefore, use errors occurred, especially when applying the contact cream (Emergency 1, Subtask 8). However, two participants did not apply the contact cream to the sensor but instead applied it to the contact surface of the pump. Nonetheless, Lin et al. [43] and Gasques et al. [25] suggested that verbal instructions could compensate for incorrect AR markings. In this study, we observed usage errors.

With both AR glasses, use errors occurred with the rotary push button on the ECMO console. The manufacturer has already addressed this usability deficit in a redesign of newer models. Therefore, we assume that the observed use errors are not due to the use of the AR glasses but to the ECMO console.

Overall, the SR of the two AR glasses differ only slightly. Poor ratings, especially for the *HoloLens 2*, would often be due to the ECMO console. We also suggest that use errors of the *HoloLens 2* could be reduced by adding voice control. Use errors of the *HMT-1* caused by the display are more difficult to compensate for. In summary, we recommend the *HoloLens 2* for use in intensive care because of its higher acceptance. However, the influence of voice control on the usability of the *HoloLens 2* should be investigated in future studies.

### 4.2 Survey of user acceptance

In the present study, AR achieved a high level of user acceptance. Only *perspicuity* was rated below average in the UEQ dimension, possibly because they lacked experience in using AR glasses (see 3.1). Veazey et al. [63] investigated user acceptance of AR for treating patients with burns. In their study, AR was also used with *HoloLens 2,* and most of the participants who were familiar with AR rated *HoloLens 2* as intuitive. Furthermore, AR achieved good user acceptance with a SUS score between 72 and 78. Bui et al. [13] also reported high AR acceptance among clinical users. In their study, they used AR to advise nurses or paramedics to treat patients with heart attacks among others. In addition, Dinh et al. [18] showed a positive acceptance of AR in telemedicine and telementoring. Simultaneously, in a meta-analysis, Hyzy et al. [32] found that the acceptance of digital emergency applications among clinical users is generally below average.

### 4.3 Limitations

The limitation of the present study is the small sample size. Conventionally, a team of nurses and physicians from various specialties handles the treatment of patients undergoing ECMO in the ICU [50]. However, in this study, only ICU nurses tested the AR glasses. Therefore, future studies should also include physicians as potential users, as they are part of the ICU team and may require remote support in ECMO emergencies. Additionally, the small sample size (n = 10) made it difficult to detect significant differences between the AR glasses. However, the aim of this study was to evaluate the usability of AR glasses to identify usability deficits. Hence, the sample size was considered sufficient for this purpose. Studies have shown that a sample of 10 participants is sufficient to identify approximately 95 % of usability issues in a product [42], [52], [64]. Furthermore, current guidelines for the evaluation of the usability of medical devices for market approval recommend a sample size of 10 participants [34].

Another aspect is the simulation environment, which did not realistically reflect all environmental conditions. Although the test was conducted in an intensive care unit, typical environmental factors such as alarms from other medical devices or other people working at the same time were missing. In real life, there are often multiple team members working on the patient, which could affect the usability of the AR glasses. Although this limitation applies to both AR glasses, it may limit the usability of the *HMT-1* to a greater extent because it is voice controlled. Background noise from conversations or loud alarms could interfere with the voice control, reducing the efficiency of the control.

Furthermore, the evaluation of usability may have been influenced by the Hawthorne effect [47]. This indicates that participants do not behave naturally in studies, because they know that the investigator is observing them. Nevertheless, usability testing and observation is an established and validated method in the field of human-computer interaction. In the user tests in virtual reality and real life, a blood pressure monitor is used. Hinricher et al. [30] could not demonstrate any influence from the investigator. In the present study, the participants were observed with both AR glasses to ensure that the effects occurred equally.

The user acceptance survey may have been influenced by the novelty effect [38]. This indicates that the participants rated novel technologies positively and perceived them as exciting. In this study, the UEQ dimensions that describe *attractiveness*, *stimulation*, and *novelty* achieved exceptionally high results, suggesting the presence of the novelty effect. Nevertheless, based on comparable studies, we assume that user acceptance is good.

Two parameters relevant to usability that were not examined in this study are learnability and time on task. As the participants performed identical tasks with both AR glasses, a learning effect may have occurred that improved performance with the second device. However, owing to the different operating concepts of the two sets of glasses, this effect is likely to primarily affect the interaction with the ECMO. As the learning effect could, therefore, strongly distort the processing times of the emergencies, the time on task was not recorded. Future studies should investigate both parameters once the identified usability issues have been resolved.

Finally, only the ICU nurses' perspective was examined in this study. However, a comprehensive evaluation of the usability of AR-based remote support for ECMO also requires the involvement of perfusionists. Their perspective is currently being investigated in another research project. Moreover, the act of having a perfusionist communicate with the bedside nurse via AR could potentially delay the arrival of the perfusionist at the bedside in case of an emergency. One possible solution to this limitation would be to assign two perfusionists on call. One dedicated to remote communication via AR and another available to respond immediately on site in the ICU.

## 5 Conclusion

This study showed that both the *HMT-1* and *HoloLens 2* were successful in assisting ICU nurses. However, use errors still occurred when making calls, which could be critical in intensive care medicine. Hence, these systems should be better adapted to the context of use to enable the efficient use of AR in intensive care. A user-centered development process is suitable for this purpose [36]. Owing to the small differences in SR, the presumed better preventability of use errors and the participants’ ratings, we prefer the *HoloLens 2* for assisting ICU nurses in troubleshooting ECMO emergencies. Furthermore, the results indicated good user acceptance in the examined user group.

## Author statement

All authors have read and approved the final version of the manuscript and agree to be accountable for all aspects of the work.

## CRediT authorship contribution statement

**Claus Backhaus:** Writing – review & editing, Supervision, Conceptualization. **Niels Hinricher:** Writing – review & editing, Investigation, Conceptualization. **Simon König:** Writing – original draft, Visualization, Methodology, Investigation, Conceptualization.

## Ethics statement

This study was conducted in accordance with the local legislation and institutional requirements. All participants provided their written informed consent to participate in this study.

## Funding

This work was supported by the Supported by the Open Access Publication Fund of FH Münster University of Applied Sciences.

## Declaration of Competing Interest

The authors declare that they have no known competing financial interests or personal relationships that could have appeared to influence the work reported in this paper.
